# Mitochondrial damage and senescence phenotype of cells derived from a novel frataxin G127V point mutation mouse model of Friedreich's ataxia

**DOI:** 10.1242/dmm.045229

**Published:** 2020-07-27

**Authors:** Daniel Fil, Balu K. Chacko, Robbie Conley, Xiaosen Ouyang, Jianhua Zhang, Victor M. Darley-Usmar, Aamir R. Zuberi, Cathleen M. Lutz, Marek Napierala, Jill S. Napierala

**Affiliations:** 1 Department of Biochemistry and Molecular Genetics, University of Alabama at Birmingham, 1825 University Boulevard, Birmingham, AL 35294, USA; 2 Department of Pathology, University of Alabama at Birmingham, 901 19th Street South, Birmingham, AL 35294, USA; 3 Center for Free Radical Biology, University of Alabama at Birmingham, Birmingham, AL 35294, USA; 4 Mitochondrial Medicine Laboratory, University of Alabama at Birmingham, Birmingham, AL 35294, USA; 5 Department of Veteran Affairs Medical Center, Birmingham, AL 35294, USA; 6 The Rare and Orphan Disease Center, JAX Center for Precision Genetics, 600 Main Street, Bar Harbor, ME 04609, USA

**Keywords:** Friedreich's ataxia, Senescence, Mitochondria, Frataxin, Point mutation, Oxidative stress

## Abstract

Friedreich's ataxia (FRDA) is an autosomal recessive neurodegenerative disease caused by reduced expression of the mitochondrial protein frataxin (FXN). Most FRDA patients are homozygous for large expansions of GAA repeat sequences in intron 1 of *FXN*, whereas a fraction of patients are compound heterozygotes, with a missense or nonsense mutation in one *FXN* allele and expanded GAAs in the other. A prevalent missense mutation among FRDA patients changes a glycine at position 130 to valine (G130V). Herein, we report generation of the first mouse model harboring an Fxn point mutation. Changing the evolutionarily conserved glycine 127 in mouse Fxn to valine results in a failure-to-thrive phenotype in homozygous animals and a substantially reduced number of offspring. Like G130V in FRDA, the G127V mutation results in a dramatic decrease of Fxn protein without affecting transcript synthesis or splicing. Fxn^G127V^ mouse embryonic fibroblasts exhibit significantly reduced proliferation and increased cell senescence. These defects are evident in early passage cells and are exacerbated at later passages. Furthermore, increased frequency of mitochondrial DNA lesions and fragmentation are accompanied by marked amplification of mitochondrial DNA in Fxn^G127V^ cells. Bioenergetics analyses demonstrate higher sensitivity and reduced cellular respiration of Fxn^G127V^ cells upon alteration of fatty acid availability. Importantly, substitution of Fxn^WT^ with Fxn^G127V^ is compatible with life, and cellular proliferation defects can be rescued by mitigation of oxidative stress via hypoxia or induction of the NRF2 pathway. We propose Fxn^G127V^ cells as a simple and robust model for testing therapeutic approaches for FRDA.

## Abstract (precis)

**Summary:** Sole expression of the mutant frataxin G127V protein is sufficient for viability of the first Friedreich's ataxia point mutation mouse model, but induces premature senescence in isolated cells.

## INTRODUCTION

Friedreich's ataxia (also called FA or FRDA) is an autosomal recessive disease with an estimated prevalence of 2-4 per 100,000 individuals (Leone et al., 1990; Winter et al., 1981). Patients develop progressive ataxia of all four limbs, associated with cerebellar dysarthria, absent reflexes in the lower limbs, sensory loss and abnormal pyramidal signs. Additionally, some patients develop optic atrophy, sensorineural hearing loss, diabetes mellitus, foot deformity, scoliosis and hypertrophic cardiomyopathy (Filla et al., 1996; Harding, 1981). Currently, there is no effective treatment.

FRDA is caused in the majority (96%) of cases by hyperexpansion of trinucleotide GAA repeats located in the first intron of the frataxin (*FXN*) gene on both alleles. The remaining (4%) of the patients are compound heterozygotes for GAA expansion on one *FXN* allele and a point mutation on the other (Cossee et al., 1999; Durr and Brice, 1996; Filla et al., 1996; Galea et al., 2016). Although not affecting the coding sequence, GAA repeats impede transcription, leading to low mRNA and protein levels. Regarding point mutations on the protein coding sequence, ≥30 pathogenic point mutations have been identified (Clark et al., 2019; Cossee et al., 1999; Galea et al., 2016), and only FXN R165C has been identified in individuals in a homozygous state (Candayan et al., 2020). Many of the point mutations affect *FXN* mRNA expression or the initiation of translation and result in more severe clinical presentations when compared with classic repeat expansion patients (Bidichandani et al., 1997; Cavadini et al., 2000b; Galea et al., 2016; Santos et al., 2010). Interestingly, a prevalent point mutation in FRDA, a single nucleotide change (c.389G>T) resulting in a missense substitution at amino acid 130 of glycine to valine (G130V), is associated with a milder clinical presentation and slower disease progression than most FRDA cases arising from homozygous GAA repeat expansions (Bidichandani et al., 1997; Clark et al., 2017; McCabe et al., 2000; Puccio and Koenig, 2000). Symptoms common for homozygous repeat expansion FRDA, including dysarthria, loss of tendon reflexes and ataxia, are not typically observed in G130V individuals, although they do distinctively exhibit an early onset spastic gait and increased prevalence of optic disk pallor and diabetes (Cossee et al., 1999; Galea et al., 2016). This suggests that the frataxin G130V (FXN^G130V^) protein is biologically active and contributes to a unique pathological mechanism.

Frataxin is a nuclear-encoded mitochondrial protein that is highly conserved throughout evolution, with homologs in eukaryotes including mammals, invertebrates and yeast (Dhe-Paganon et al., 2000). Complete absence of wild-type Fxn (Fxn^WT^) is incompatible with life, because *Fxn* null mice die early during embryogenesis (Cossee et al., 2000), and mouse embryonic fibroblasts (MEFs) lacking Fxn^WT^ fail to divide (Calmels et al., 2009). After translation in the cytoplasm, frataxin is transported into the mitochondria using an N-terminal targeting sequence. Upon entry, it undergoes a two-step proteolytic cleavage by mitochondria processing peptidase (MPP) to generate the mature protein (Koutnikova et al., 1998). It has been reported that the G130V mutation modulates interaction with MPPβ and affects the maturation process (Cavadini et al., 2000a). In fact, the ratio of intermediate to mature frataxin protein is increased in fibroblasts from FRDA G130V patients, suggesting an impairment in maturation processing (Clark et al., 2017). The G130V mutation has been found to destabilize the protein structure, leading to low levels of mature frataxin in yeast (Cavadini et al., 2000a).

Despite the availability of numerous disease models, no single model perfectly mimics the human FRDA state. Several FRDA mouse models have been generated thus far, but all of them aimed to recapitulate a condition of low frataxin expression by engineering long tracts of the expanded GAAs (Miranda et al., 2002; Pook et al., 2001), conditional deletion of Fxn (Puccio et al., 2001) or transcript depletion (short hairpin RNA and small interfering RNA) (Chandran et al., 2017). Thus far, no animal models have been reported that specifically analyze pathological consequences of frataxin point mutations.

To determine the impact of the G130V mutation *in vivo*, we generated mice harboring the homologous mutation in murine frataxin (G127V). Our studies indicate that in the absence of Fxn^WT^, expression of the Fxn^G127V^ protein is sufficient for viability, although significant postnatal lethality was observed. Moreover, cells derived from homozygous mutant embryos (*Fxn^G127V/G127V^*; MUT) demonstrate a significant increase in a senescent phenotype, significant mitochondrial damage and reduced proliferative capacity that can be rescued after incubation in a hypoxic environment or treatment with the nuclear factor erythroid 2 (NF-E2)-related factor 2 (NRF2) activators dimethylfumarate (DMF) or omaveloxolone (RTA 408), conditions and compounds that mitigate oxidative stress (La Rosa et al., 2020; Saidu et al., 2019; Wong et al., 1999). This new cellular model could be considered as a screening platform for testing the efficacy of small molecules and human *FXN* or FXN replacement therapies aimed to alleviate the effects of frataxin deficiency.

## RESULTS

### Generation of frataxin G127V knock-in mice

**Fig. 1.**
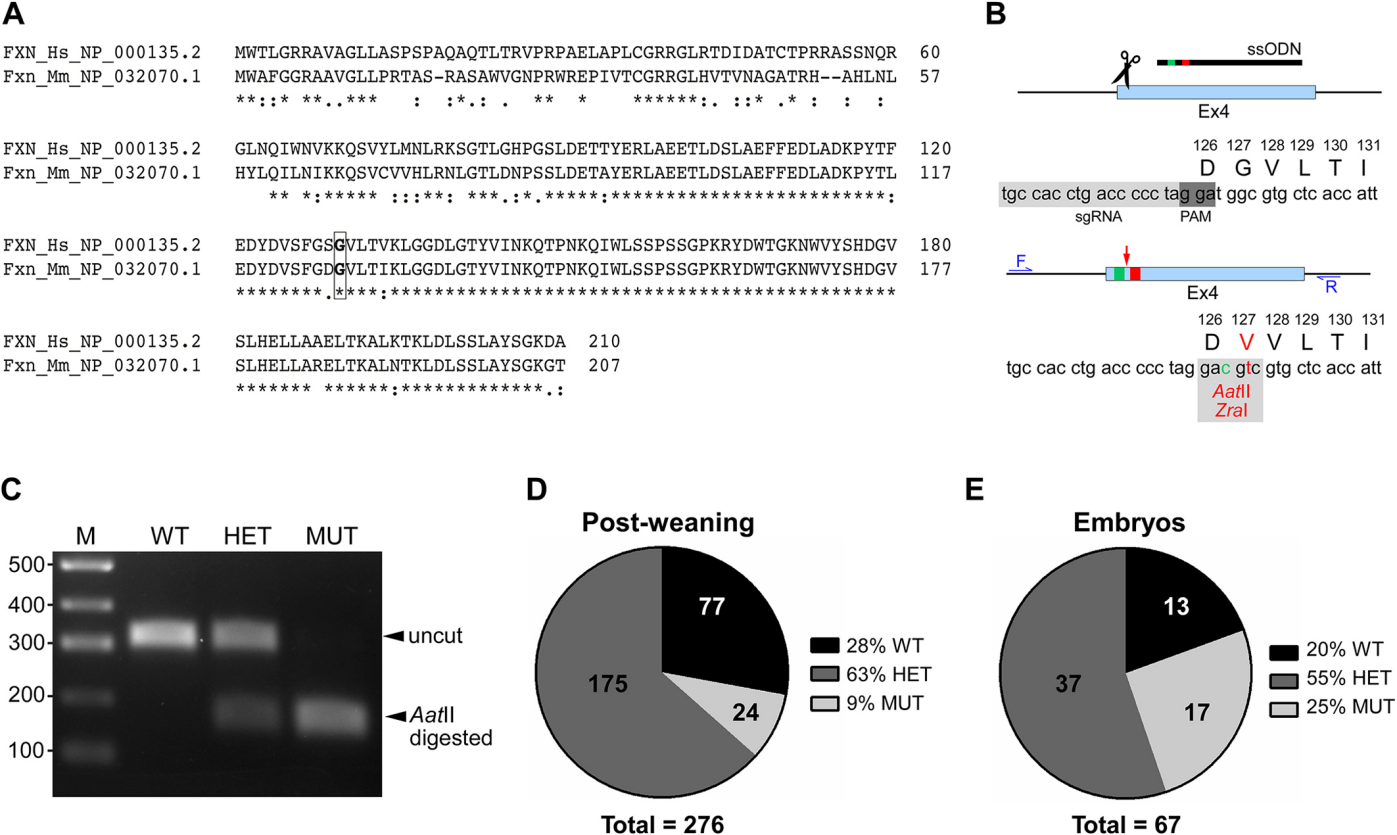
**Generation of** **Fxn^G127V^** **mice via CRISPR/Cas9-mediated knock-in strategy.** (A) Alignment of human and mouse frataxin amino acid sequences, with the position of glycine 130/127 in bold type and surrounded by a box. (B) Sequence of the edited region of the *Fxn* gene: scissors illustrate location of DNA break in exon 4 of *Fxn*; gray box indicates position of single guide RNA (sgRNA); arrow indicates location of missense mutation (red box) and silent mutation (green box). The result of the modification is G→V substitution and creation of an AatII restriction site. F and R illustrate locations of genotyping primers. The abbreviation ssODN refers to the single-stranded oligodeoxynucleotide donor template. PAM, protospacer adjacent motif. (C) Representative agarose gel electrophoresis image showing restriction fragment length polymorphism genotyping products for WT, HET and MUT cells. (D) Observed frequency of genotype distribution at weaning is plotted, with the number of animals per group indicated on each pie chart; *n*=276 biological replicates. (E) The observed frequency of the genotype distribution of embryos at 20 dpc is plotted, with the number of animals per group indicated on each pie chart; *n*=67 biological replicates.

Human and mouse mature frataxin proteins share 71.5% amino acid sequence identity (CLUSTALW; 73% by BLASTP), indicating that the outcome of the amino acid change is comparable between the two (Fig. 1A). To study the effects of the disease-causing point mutation on the endogenous Fxn protein *in vivo*, we used CRISPR/Cas9 to introduce the (c.401G>T) mutation in exon 4 of murine *Fxn* (Fig. 1B). The resulting G127V substitution is equivalent to the human pathogenic G130V missense mutation. Additionally, a silent mutation (c.399T>C) was introduced to serve as a Cas9 blocking mutation and to create a new restriction site (AatII/ZraI) for genotyping (Fig. 1C).

After CRISPR/Cas9 editing, the integrity of the *Fxn* coding sequence and the incorporation of the G127V point mutation were verified by sequencing complementary DNA generated from cerebella of two mice heterozygous for the *Fxn^G127V^* allele (*Fxn^G127V/+^*). The results from both animals indicated the presence of only the two engineered point mutations, with the remaining *Fxn* coding sequence being free of errors. Inter-crossing of *Fxn^G127V/+^* mice resulted in progeny of all possible genotypes: WT, mice homozygous for wild-type frataxin (*Fxn^+/+^*; *n*=77; 28%); HET, mice heterozygous for *Fxn^+^* and *Fxn^G127V^* alleles (*Fxn^G127V/+^*; *n*=175; 63%); and MUT, mice homozygous for the *Fxn^G127V^* allele (*Fxn^G127V/G127V^*; *n*=24; 9%). MUT mice were viable but were under-represented postweaning (9% instead of the expected 25%; Fig. 1D). However, reduced representation of MUT animals was not detected at the late embryonic stage, 20 days postcoitum (dpc) (Fig. 1E; Fig. S1), suggesting that MUT mice develop normally *in utero* but fail to thrive shortly after birth. Notably, late-stage MUT embryos were significantly underweight compared with their WT and HET counterparts, suggesting a disadvantage for competitive survival after birth (Fig. S1). Furthermore, mating of HET to WT (C57BL/6J) did not result in a significant difference in mutant allele transmission (*P*=0.074; Table S1).

Owing to the low number of MUT animals immediately available for study, we conducted molecular analyses on effects of the G127V mutation on Fxn expression and function using MEFs, because they could readily be isolated and cultured *ex vivo*. This homogeneous cellular model allows, for the first time, in-depth analyses of the endogenous Fxn^G127V^ protein in the absence of Fxn^WT^.

### Endogenous Fxn protein levels are markedly reduced by the G127V mutation

**Fig. 2.**
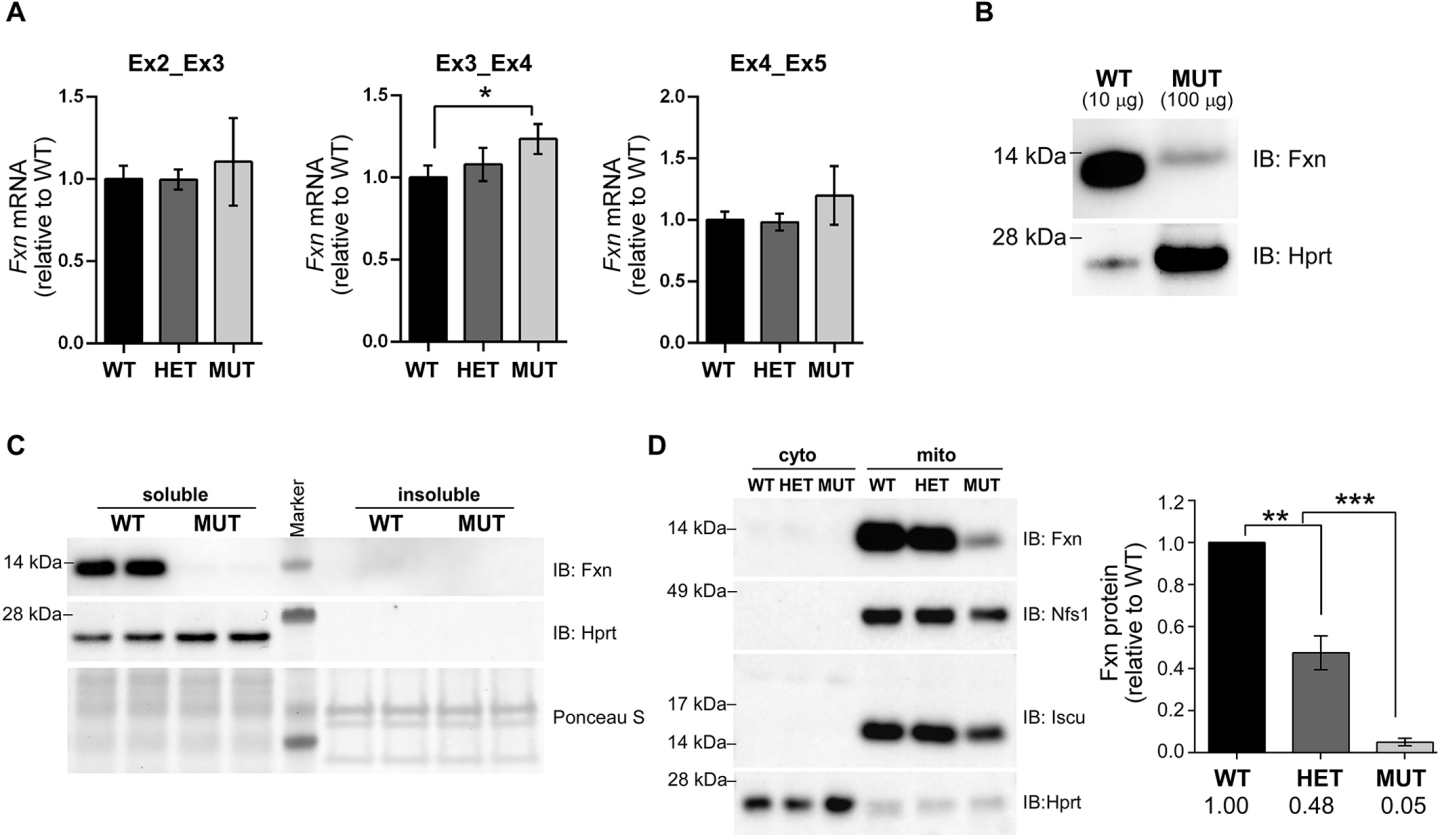
***Fxn^G127V^* mRNA expression is not correlated with immunodetectable protein levels.** (A) Frataxin mRNA expression levels measured by real-time RT-qPCR using RNA extracted from WT, HET and MUT MEFs. *Fxn* transcripts were normalized to *Gapdh* and plotted relative to WT samples. Data are shown for three different primer pairs used for analyses: Ex2_Ex3 (spanning exons 2 and 3; upstream of the mutation); Ex3_Ex4 (spanning exons 3 and 4; encompassing the mutation site); and Ex4_Ex5 (spanning exons 4 and 5; downstream of the mutation). Bars show the mean±s.d. and represent data from two independent MEF lines per genotype (*n*=2 biological replicates), combined from two independent experiments; total measurements per bar=4. Student's unpaired *t*-test (*P<0.05). (B) Frataxin protein expression levels in WT and MUT MEFs was determined by western blot. Fxn^G127V^ protein can be detected only with enhanced sensitivity western blotting techniques. To avoid oversaturation of the signal, 10 μg of WT lysate and 100 μg of MUT lysate were loaded per lane. The blot shown is representative of three experiments performed with two independent MEF lines per genotype (*n*=2 biological replicates); total measurements=6. (C) Western blot analysis of protein samples fractionated to soluble and insoluble fractions is shown, with Hprt and Ponceau S staining serving as loading controls. The blot shown is representative of two experiments performed with two independent MEF lines per genotype (*n*=2 biological replicates); total measurements=4. (D) A representative western blot showing mitochondria-enriched fractions prepared from WT, HET and MUT MEFs. Fifty micrograms of each fraction was loaded per lane. Hprt serves as a positive control for the cytosolic fraction, whereas Nfs1 and Iscu are positive controls for the mitochondrial fraction. The blot shown is representative of two experiments performed with two independent MEF lines per genotype (*n*=2 biological replicates). Quantitative values are plotted as the mean±s.d. (total measurements per bar=4), with significant differences determined using ordinary one-way ANOVA (**P<0.01, ***P<0.001). IB, immunoblot.

To determine how the mutation affected Fxn expression, we first measured the levels of *Fxn* mRNA and Fxn protein in MEF cell lines established from WT, HET and MUT embryos. Analyses by quantitative reverse transcription PCR (RT-qPCR) demonstrated that the G127V mutation did not decrease *Fxn* mRNA expression (Fig. 2A). In addition, because glycine 127 is the second amino acid of exon 4, we tested whether the nucleotide substitutions introduced near the 5′ end of this exon affected splicing of the *Fxn* transcript. RT-qPCR results using primer pairs designed upstream, spanning and downstream of the targeted site revealed that introduction of the mutations did not decrease steady-state levels of the *Fxn* transcript (Fig. 2A). Moreover, RT-qPCR products spanning the *Fxn* exon 3-4 junction were amplified from MUT mRNA, cloned and sequenced, and the results (5/5 clones) demonstrated no abnormally spliced transcripts. Taken together, analyses of *Fxn* mRNA from WT, HET and MUT MEFs indicated that introduction of the G127V mutation did not decrease production or negatively affect splicing of *Fxn* transcripts.

Next, we turned our attention to how the G127V mutation impacted Fxn protein levels, because we have previously reported greatly reduced total Fxn levels, especially of the mature (M) isoform, in fibroblasts derived from FRDA G130V patients (Clark et al., 2017). We first tested three distinct, commercially available antibodies using lysates prepared from HEK-293T cells transiently expressing exogenous murine Fxn^WT^ or Fxn^G127V^ proteins to address the possibility of epitope masking by the point mutation (Fig. S2A). Albeit at much lower levels than Fxn^WT^, the Fxn^G127V^ protein was detected by both FXN polyclonal antibodies (first and second panels). However, as previously reported (Koutnikova et al., 1998), the FXN monoclonal antibody (clone 1G2) failed to recognize the mutant protein (third panel). Western blotting of whole cell lysates revealed that Fxn^G127V^ protein levels were profoundly decreased in MUT compared with HET or WT MEFs to such an extent that western blot techniques with enhanced sensitivity were necessary to detect the Fxn^G127V^ protein in whole cell lysates (Fig. 2B,C). Similar results were obtained with the other FXN polyclonal antibody tested (Fig. S2B; GTX54036).

In light of the low western blot signal from MUT samples, we examined the possibility of Fxn^G127V^ protein aggregation. As evident from analysis of insoluble fractions of whole cell lysates, neither Fxn^WT^ nor Fxn^G127V^ was found to aggregate (Fig. 2C). Finally, despite lower immunodetectable levels, the Fxn^G127V^ protein was exclusively found in mitochondria-enriched fractions, in a similar manner to Fxn^WT^ protein (Fig. 2D). Western blot quantitation revealed that Fxn^G127V^ levels in MUT mitochondrial extracts were only 5% of those observed for Fxn^WT^ protein in WT mitochondrial extracts (Fig. 2D). These results indicated that steady-state levels of immunodetectable Fxn^G127V^ protein were below the levels of Fxn^WT^ protein measured in human FRDA cases caused by homozygous repeat expansions (10-30% of levels in healthy people) (Deutsch et al., 2010; Long et al., 2017; Nachbauer et al., 2011; Saccà et al., 2013) and other FRDA mouse models [e.g. knock-in/knockout (KIKO) mice (25-36% of WT) (Miranda et al., 2002; Perdomini et al., 2013)]. Nevertheless, this low amount of Fxn^G127V^ protein was sufficient for MEF survival and mouse development.

### Cells expressing only Fxn^G127V^ exhibit reduced proliferation

**Fig. 3.**
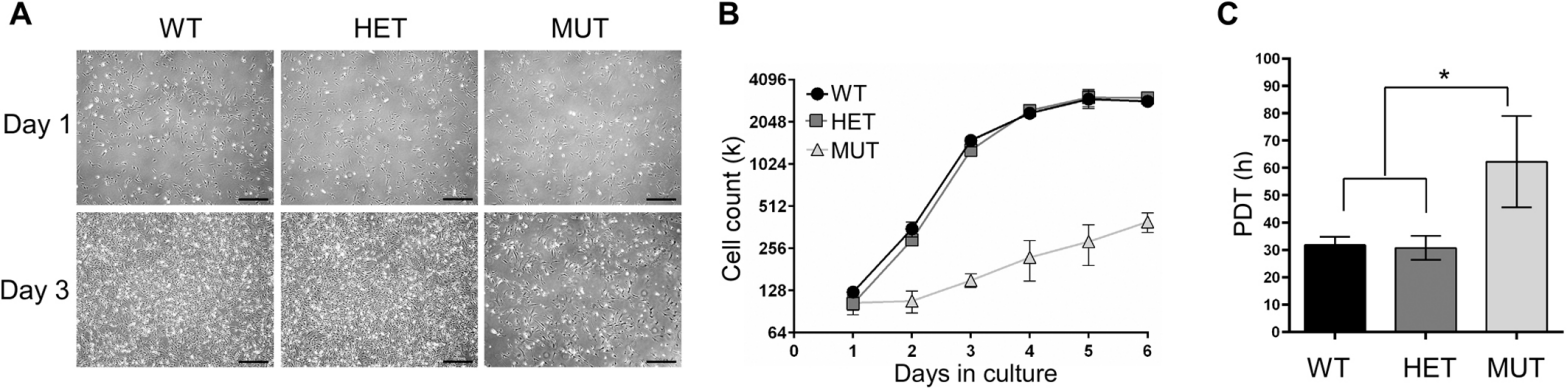
**Fxn^G127V^ MUT MEFs exhibit slow growth in culture.** (A) Representative phase-contrast images of WT, HET and MUT MEFs at days 1 and 3 in culture after equal plating. Three independent growth curve experiments were performed using two independent MEF lines per genotype (*n*=2 biological replicates). Scale bars: 500 μm. (B) Growth analysis of WT, HET and MUT MEFs over 6 days in culture. Cells (at passage 3) were seeded at equal densities in duplicate wells at day 0, then counted every 24 h. The experiment was repeated three times using two independent MEF lines per genotype (*n*=2 biological replicates), and a representative curve is shown as the mean±s.d. (C) WT, HET and MUT MEF population doubling times as calculated from the growth curves (B). Bars show the mean±s.d. calculated from three independent growth curve experiments; total measurements per bar=3. Significant differences were determined using ordinary one-way ANOVA (*P<0.05).

We next turned our attention to overt cellular phenotypes observed while establishing and culturing independent batches of MUT, HET and WT MEF cell lines. To measure growth rates of lines representing the three genotypes, equal numbers of cells were seeded, and the cells were counted every 24 h over a 6-day period. We consistently noted slower proliferation of Fxn^G127V^ homozygous MUT MEFs when compared with WT and HET cell lines that were established in parallel (Fig. 3A,B). Calculated population doubling times (PDTs) of WT, HET and MUT MEFs from three independent experiments were 31.8, 30.7 and 62.3 h, respectively (Fig. 3C), which is a 2-fold slower growth rate of MUT MEFs when compared with WT or HET.

**Fig. 4.**
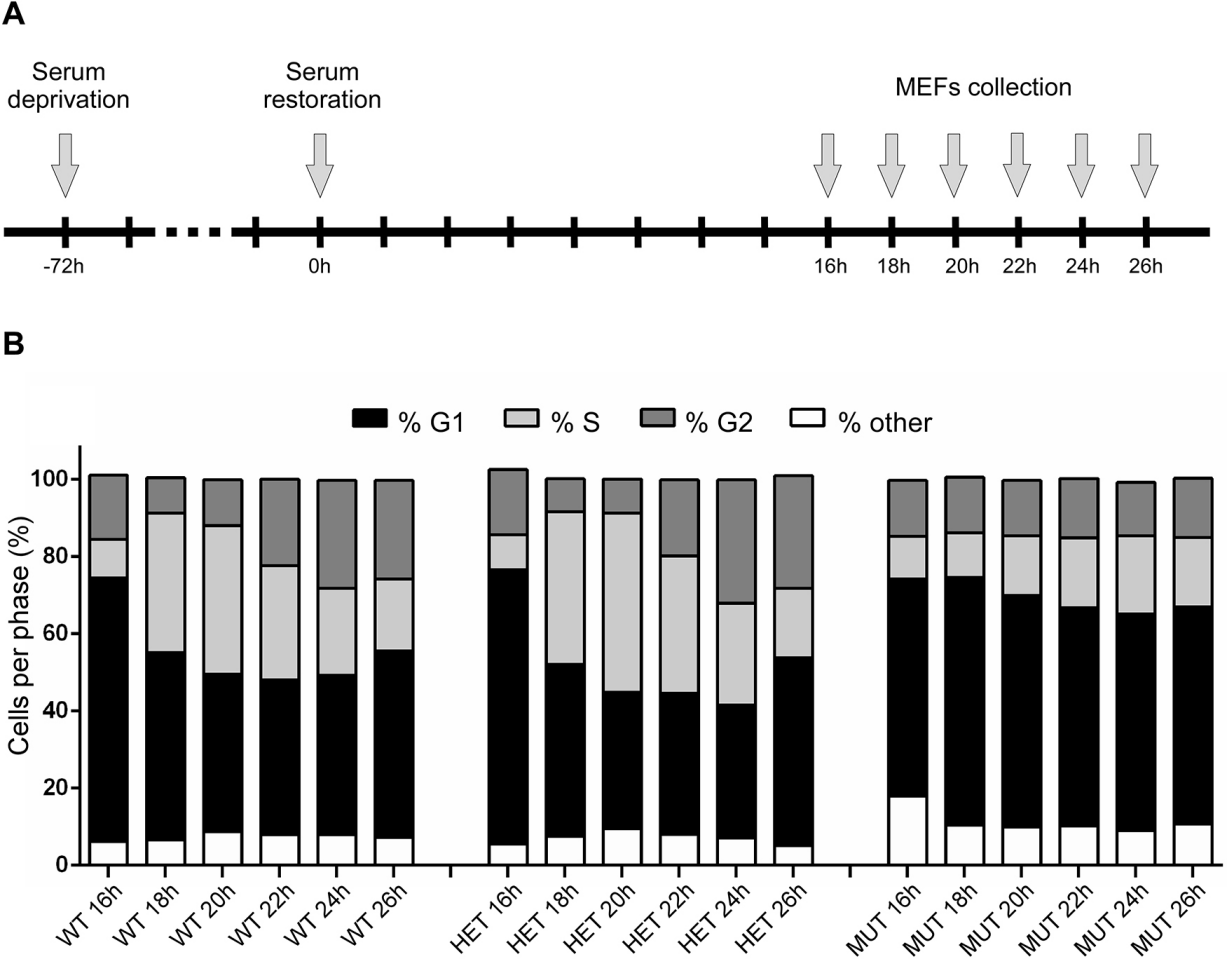
**Fxn^G127V^ MUT MEFs proliferate more slowly after cell cycle synchronization.** (A) A schematic representation of the cell cycle synchronization protocol is shown, whereby quiescence is induced by serum deprivation for 72 h, followed by serum restoration and progression into the cell cycle. (B) Flow cytometry analysis of cell cycle distribution of WT, HET and MUT MEFs (at passage 4) monitored from 16 to 26 h after serum restoration. Each time point is an average of two replicates.

To test whether the diminished growth rate of MUT MEFs was attributable to slower progression through the cell cycle, we analyzed their distribution between G1, S and G2 phases using flow cytometry. In synchronized cultures, a smaller percentage of MUT cells were actively dividing compared with WT or HET MEFs (Fig. 4A,B). The WT and HET MEFs entered S phase ∼18 h after serum supplementation, whereas MUT MEFs mostly remained quiescent beyond 26 h (the last measurement taken). These results aligned with the calculated PDTs (Fig. 3C) and demonstrated a profound proliferation defect in cells expressing a low level of Fxn^G127V^ protein in the absence of Fxn^WT^.

### Fxn^G127V^ MUT MEFs exhibit increased senescence

**Fig. 5.**
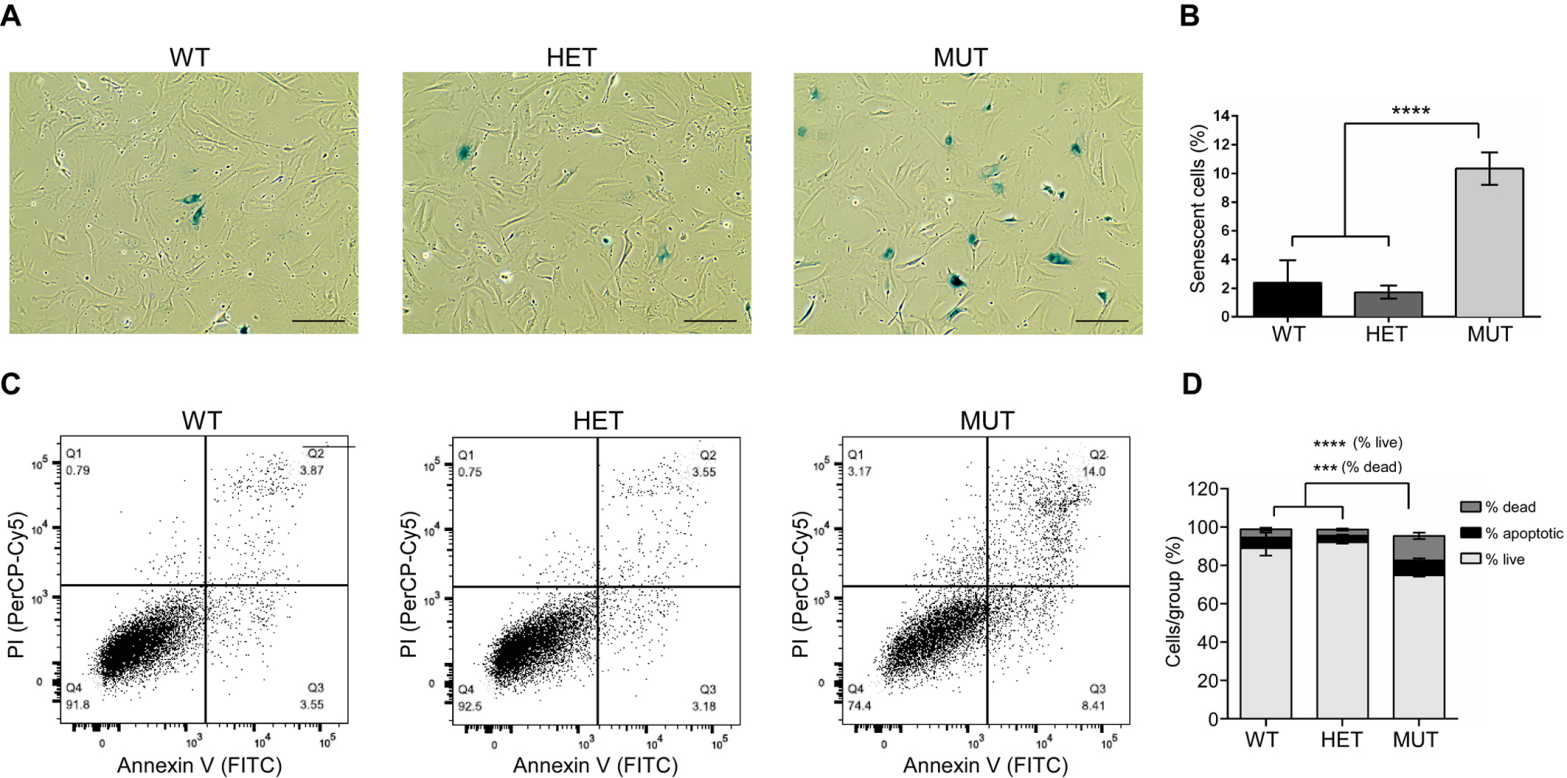
**Fxn^G127V^ MUT MEFs are prone to senescence and cell death.** (A) Representative phase-contrast images of WT, HET and MUT MEFs stained for detection of SA-β-galactosidase (blue cells) taken at ×200 total magnification. Scale bars: 200 μm. (B) Quantification of senescent cells in WT, HET and MUT MEF cultures is plotted as a percentage of total cells counted. Each bar represents the mean±s.d. of six independent fields, in which ≥125 cells were counted per field; *n*=2 biological replicates, total measurements per bar=6. A significant difference between the MUT group and the WT and HET groups was determined by ordinary one-way ANOVA (****P<0.0001). (C) Representative scatter plots are shown for flow cytometry analyses after annexin V and propidium iodide (PI) staining of WT, HET and MUT MEFs. The histograms illustrate the number of cells stained with PI (*y*-axis) and/or annexin V (*x*-axis), and the populations are divided into quadrants (Q1-Q4). Ten thousand events were collected for each measurement, and measurements were repeated in two independent experiments with two technical replicates per experiment; *n*=2 biological replicates, total measurements=4. (D) The percentages of live (Q4), apoptotic (Q3) and dead (Q2) cells within WT, HET and MUT MEF cultures were averaged from two independent annexin V/PI flow cytometry experiments; *n*=2 biological replicates; total measurements per bar=4. Significant differences were determined between MUT and WT, HET groups by two-way ANOVA using Tukey's method for multiple comparisons (***P<0.001, ****P<0.0001). Cells used for all staining experiments were early passage (passage 3 or 4).

In addition to suppression of the cell cycle, we considered additional processes that could negatively impact MUT cell numbers in culture (Fig. 3). Given that most of the MUT cell population appeared to be arrested in the G1 phase (Fig. 4B), we turned our attention first to cellular senescence. It was previously shown that acute and severe depletion of FXN (∼80% reduction) in immortalized cells resulted in inhibited proliferation, accompanied by increased G1 phase arrest and cellular senescence (Bolinches-Amoros et al., 2014). To test whether MUT MEFs, expressing low levels of Fxn^G127V^, were prone to a similar fate, we examined induction of senescence by detection of senescence-associated β-galactosidase (SA-β-gal) activity, a known characteristic of senescent cells (Dimri et al., 1995). As expected, WT and HET MEF cultures were nearly devoid of senescent cells, with only 2.4±1.6% and 1.7±0.5% of the cultures staining positive for SA-β-gal activity, respectively (Fig. 5A,B). In contrast, cells positive for SA-β-gal activity represented 10.3±1.1% in MUT MEF cultures, indicating a ∼4.3-fold increase in senescence induction in MUT MEFs compared with WT (Fig. 5B). Moreover, we found that MUT MEFs senesced at a significantly faster rate than WT cells when compared at each passage (Fig. S3).

In addition to senescence, acute frataxin depletion has been shown to induce cell death via apoptosis (Loría and Diáz-Nido, 2015; Mincheva-Tasheva et al., 2014; Palomo et al., 2011). To determine whether MUT MEFs were predisposed to apoptosis, we quantified the percentage of viable, apoptotic and dead cells in actively dividing cultures using flow cytometry. WT and HET MEF populations were composed mostly of viable cells (89±4% and 92±0.7%, respectively), whereas the percentage of viable cells in MUT MEF populations was significantly lower (75±0.8%; *P*<0.0001) (Fig. 5C,D). By contrast, the percentage of dead cells [propidium iodide (PI) and annexin V positive] was significantly higher for MUT populations (12.8±1.7%) compared with WT (4.5±0.8%; *P*<0.001) or HET (3.3±0.5%; *P*<0.001). Finally, although the proportion of apoptotic cells was higher in MUT than in WT and HET MEF populations, the difference did not reach statistical significance (MUT, 7.7±1.1%; WT, 5.5±2.7%; and HET, 3.5±0.4%). Taken together, these data demonstrated that in the absence of Fxn^WT^, expression of Fxn^G127V^ was sufficient for survival, but that murine cells expressing only the mutant protein had reduced proliferative capacity and augmented cell death and senescence.

### Mitochondrial integrity in Fxn^G127V^ MUT MEFs decreases over time

**Fig. 6.**
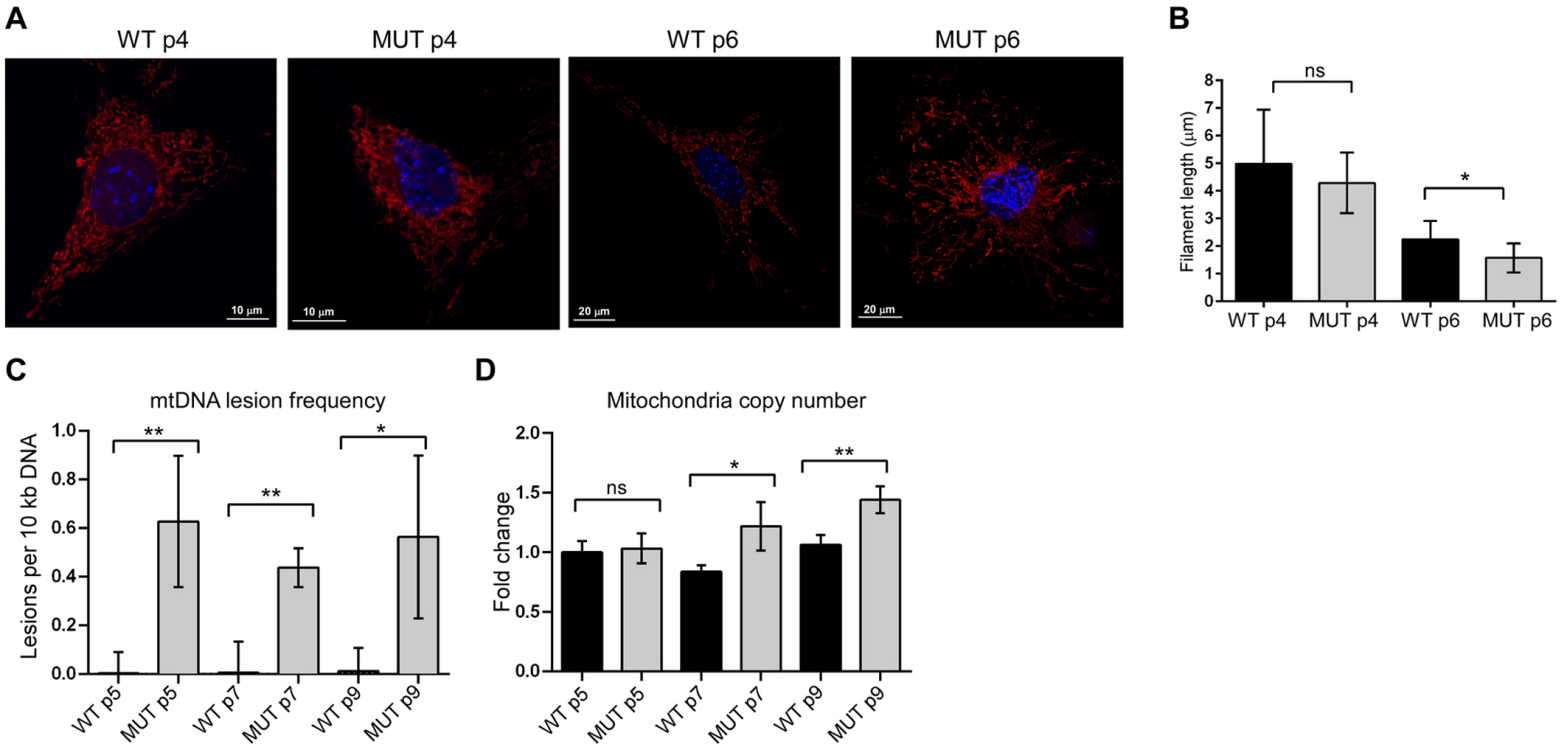
**Increased mitochondrial damage in Fxn^G127V^ MUT MEFs.** (A) Representative confocal images of WT and MUT passage (p) 4 and 6 MEFs stained with MitoTracker DeepRed FM are shown (*z*-stack maximum intensity projections). Cells were imaged using an oil immersion ×63 objective. Three independent experiments were conducted (staining and imaging) using two independent MEF lines per genotype (*n*=2 biological replicates). (B) Fields from ten images per group (A) were analyzed, and mitochondrial network filament lengths were calculated from an average of 415 measurements per field using IMARIS Filament Tracer software. Each bar represents the mean±s.d. of the averaged measurements per field; total averaged measurements per bar=6-9. The significant difference between MUT and WT filament lengths was determined by Student's unpaired *t*-test (*P<0.05). (C) Relative quantitation of mitochondrial DNA lesions in WT and MUT MEFs calculated from the ratio of long PCR (10 kb) product normalized to the short PCR (117 bp) product is plotted as the mean±s.d; *n*=2 biological replicates, total measurements per bar=4. Significant differences were determined by Student's unpaired *t*-tests (*P<0.05, **P<0.01). (D) Relative quantitation of mitochondrial DNA copy number normalized to genomic DNA is plotted as the mean±s.d; *n*=2 biological replicates, total measurements per bar=4. Significant differences were determined by Student's unpaired *t*-tests (ns, not significant; *P<0.05, **P<0.01).

Considering that consequences of frataxin deficiency are acutely observed in mitochondria (Stepanova and Magrané, 2020), we sought to evaluate effects of Fxn^G127V^ expression on functions of this organelle. Increased mitochondrial fragmentation has been reported for some neuronal subtypes derived from FRDA mouse models (Lin et al., 2017b; Mollá et al., 2017), and fragmentation in FRDA patient cells becomes apparent after an external oxidative insult (Lefevre et al., 2012). To assess mitochondrial morphology and network integrity in WT and MUT MEFs, cells were labeled with MitoTracker Deep Red and imaged. As shown in Fig. 6A, mitochondrial networks were not qualitatively different between the cell lines at early passages, but fragmented networks were observed more frequently in MUT MEFs at late passages compared with those in WT cells. Measurement of the length of mitochondria with IMARIS Filament Tracer software revealed shorter filament lengths in late passage MUT cells (Fig. 6B).

Frataxin deficiency is also linked with mitochondrial DNA (mtDNA) damage (Haugen et al., 2010; Karthikeyan et al., 2003). Indeed, our previous study in FRDA fibroblasts found that low FXN levels led to increased mtDNA damage and decreased mtDNA repair capacity (Bhalla et al., 2016). To determine whether the integrity of mtDNA was compromised in cells expressing only Fxn^G127V^, we analyzed mtDNA isolated from WT and MUT MEFs using a quantitative PCR assay based on the principle that DNA lesions can slow down or block the progression of DNA polymerase (Bhalla et al., 2016; Ponti et al., 1991; Redmann et al., 2018). This type of assay detects various forms of DNA damage, from single- or double-strand DNA breaks and gaps to specific chemical lesions (Lehle et al., 2014). Quantitation of long (10,085 bp) and short (117 bp) mtDNA fragments amplified from WT and MUT MEFs revealed an increase in lesion frequency in cells expressing only Fxn^G127V^ (Fig. 6C), consistent with a higher level of mtDNA damage.

Finally, we calculated the mtDNA copy number by comparing the amount of mtDNA with an intergenic region of nuclear DNA [fragment of the hexokinase 2 (*Hk2*) gene]. These results revealed that the mtDNA content was not changed in early passage MUT MEFs but that it increased significantly in later passage cells compared with WT MEFs (Fig. 6D). Taken together, these effects on mtDNA integrity and the number of mitochondria suggested that Fxn^G127V^ MUT MEFs accumulated defective mtDNA over time which might then contribute to the emergence of the senescence phenotype.

### Mitochondria of Fxn^G127V^ MUT MEFs exhibit altered fatty acid utilization

**Fig. 7.**
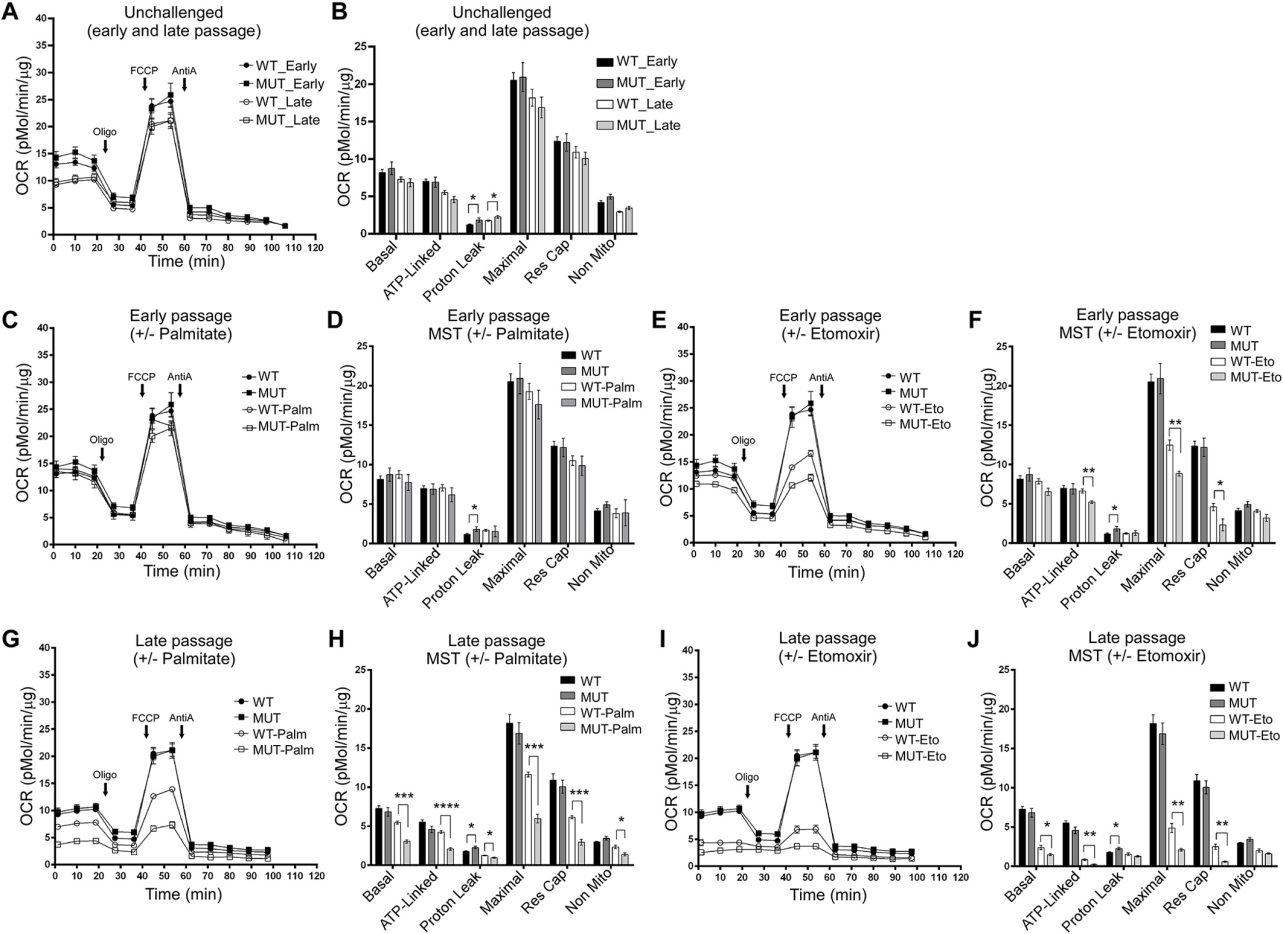
**Bioenergetic characteristics of Fxn^WT^ and Fxn^G127V^ MUT MEFs.** (A-J) Shown are OCRs and mitochondrial function indices recorded during mitochondrial stress tests conducted on the following: (A,B) early (p4) and late (p8) passage WT and MUT MEFs; (C,D) early (p4) passage WT and MUT MEFs with or without palmitate-BSA (25 µg/ml) treatment; (E,F) early (p4) passage WT and MUT MEFs with or without etomoxir (10 µM) treatment; (G,H) late passage (p8) WT and MUT MEFs with or without palmitate-BSA (25 µg/ml) treatment; or (I,J) late passage (p8) WT and MUT MEFs with or without etomoxir (10 µM) treatment. Data for unchallenged WT and MUT MEFs (A,B) are replotted on all graphs for side-by-side comparison of all treatments/conditions. Comparisons were made with Student's unpaired *t*-tests between WT and MUT MEFs for each condition. Three independent experiments were performed for panels A-J, each with at least four technical replicates per sample. Representative plots are shown for each as the mean±s.e.m.; total measurements per bar=12 (unchallenged), 4 (etomoxir) and 4 (palmitate). AntiA, antimycin A; Eto, etomoxir; FCCP, carbonyl cyanide-4 (trifluoromethoxy)phenylhydrazone; Oligo, oligomycin; Palm, palmitate. *P<0.05, **P<0.01, ***P<0.0.001, ****P<0.0001.

FXN^WT^ knockdown (KD) and overexpression both negatively impact mitochondrial energy production (Bolinches-Amoros et al., 2014; Vannocci et al., 2018). However, the effect of FXN point mutations on cellular bioenergetics has not been studied. To assess the effect of Fxn^G127V^ expression on bioenergetics, we measured cellular oxygen consumption rate (OCR) directly in living cells. Initially, we performed mitochondrial stress tests for WT and MUT MEFs in basal conditions (unchallenged; Fig. 7A,B) (Dranka et al., 2011; Hill et al., 2012). Interestingly, basal and maximal respiration rates were not different between WT and MUT MEFs at early or late passages in these conditions. However, an increase in proton leak was detected in the MUT cells (compared with WT) at both early and late passages (Fig. 7B), which is consistent with a decrease in bioenergetic efficiency, possibly related to mitochondrial membrane damage.

Next, we assessed the status of fatty acid oxidation in Fxn^G127V^ MUT MEFs. Depletion of endogenous fatty acids followed by palmitate supplementation revealed similar profiles for early passage WT and MUT MEFs, suggesting that both had the capacity to utilize exogenous fatty acids as an energy source in these test conditions (Fig. 7C,D). To determine the dependence of the cells on oxidation of endogenous fatty acids, we next treated the MEFs with etomoxir, which, at low concentrations (10 µM), blocks ∼80% of mitochondrial import of fatty acids via irreversible inhibition of carnitine palmitoyltransferase 1 (CPT1) (McGarry et al., 1991; Yao et al., 2018). After etomoxir treatment, we observed a significant reduction of maximal respiration in both WT and MUT MEFs, with a greater inhibition shown by MUT cells for both ATP-linked and maximal respiration, in addition to their reserve capacity (Fig. 7E,F). In a similar manner, we analyzed fatty acid utilization of late passage WT and MUT MEFs and observed significant decreases in OCR for both types of cells after both treatments, with exacerbated effects observed for MUT MEFs (Fig. 7G-J).

### Mitigation of oxidative stress increases viability of Fxn^G127V^ MUT MEFs

**Fig. 8.**
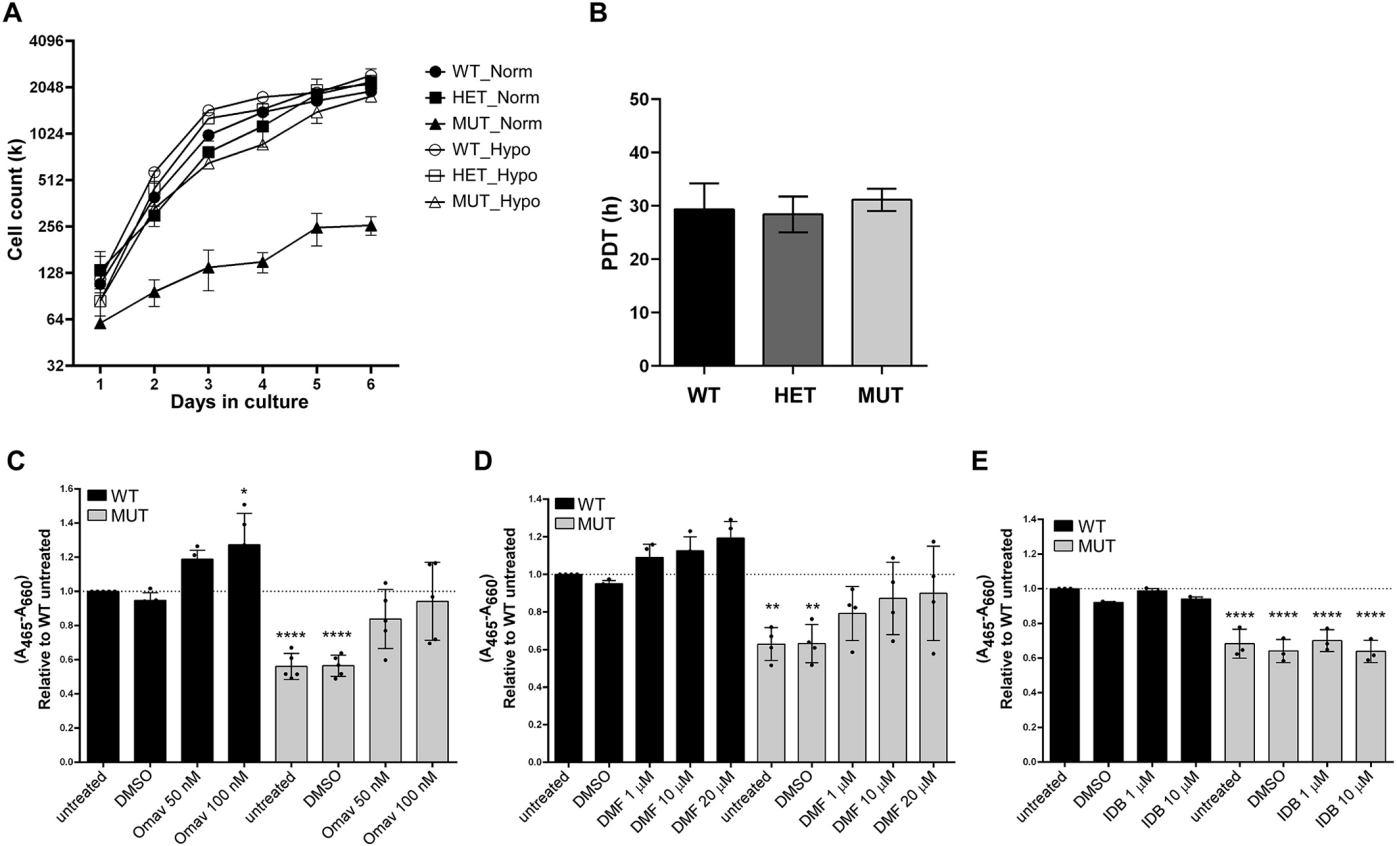
**Viability of Fxn^G127V^ MUT MEFs can be rescued by mitigating oxidative stress.** (A) Growth analysis of WT, HET and MUT MEFs grown in hypoxic conditions over 6 days in culture. Cells were seeded at equal densities in duplicate wells at day 0, then counted every 24 h. The experiment was repeated twice using two independent MEF lines per genotype (*n*=2 biological replicates), and a representative curve is plotted as the mean±s.d. (B) WT, HET and MUT MEF population doubling times as calculated from the growth curves shown in A. Bars represent the mean±s.d.; total measurements per bar=4. (C-E) XTT assays were performed after 24 h treatments of WT and MUT MEFs with respective compounds. Absorbances were recorded (specific *A*_465 nm_ and background *A*_660 nm_), and data for each treatment are expressed relative to untreated WT cells. Each bar represents the mean±s.d. of at least three independent experiments (black dots) performed using two independent MEF lines per genotype (*n*=2 biological replicates); total measurements per bar=5 (Omav), 4 (DMF) and 3 (IDB). Significant differences were determined by ordinary one-way ANOVA comparing each treatment with untreated WT (*P<0.05, **P<0.01, ****P<0.0001).

It was recently demonstrated that hypoxic conditions rescue frataxin null organisms from lethality (Ast et al., 2019). Indeed, we observed a significant increase in proliferation of MUT MEFs, rescued to rates similar to those observed for WT and HET MEFs, when the cells were cultured in a hypoxic environment (Fig. 8A). The calculated PDTs for WT, HET and MUT cells grown in hypoxic conditions were 29.4, 28.4 and 31.1 h, respectively, with no significant differences determined between the genotypes (Fig. 8B). We also used XTT assays to test the effects of several compounds shown to improve biochemical and cellular phenotypes of FRDA cell line models, including idebenone (IDB) and omaveloxolone (RTA 408; Omav), which are molecules that have been or are currently included in clinical trials for FRDA (Zhang et al., 2019), and dimethyl fumarate (Tecfidera; DMF), which is a molecule approved for treatment of multiple sclerosis (http://www.fda.gov) (Gold et al., 2012; Saidu et al., 2019). Viability of MUT MEFs was improved after 24 h of treatment with Omav (Fig. 8C) and DMF (Fig. 8D) to levels that no longer differed significantly from untreated WT cells, whereas idebenone treatment had no effect (Fig. 8E). Taken together, these results implicated oxidative stress as a driver of reduced survival and proliferation of Fxn^G127V^ MUT MEFs and suggested that restoring the redox balance via NRF2 activation significantly improved the viability of these cells.

## DISCUSSION

Herein, we report generation of mice carrying a G127V mutation in the endogenous *Fxn* locus, analogous to the pathogenic G130V mutation observed in individuals diagnosed with FRDA (Bidichandani et al., 1997). Previous studies of FXN^G130V^ in MEF cultures demonstrate that transgenic expression of the mutant human protein can rescue the lethal phenotype of *Fxn* knockout (Calmels et al., 2009). The viability of our homozygous *Fxn^G127V^* mice also demonstrates that the Fxn^G127V^ mutant protein retains a level of function that, on its own, sustains life in a whole organism. Individuals carrying homozygous *FXN* G130V mutations have not been identified. The possibility exists that homozygous *FXN* G130V mutations could result in embryonic or perinatal lethality, an idea supported, in part, by our results demonstrating reduced representation of Fxn^G127V^ MUT mice after birth. Other contributory factors could be under- or misdiagnosis owing to underutilization of high-throughput sequencing methods to support molecular diagnoses and atypical clinical presentations.

The normal Mendelian genotype distribution of mouse embryos at 20 dpc and reduced representation of MUT neonates is intriguing. MUT mice seem to develop normally *in utero*, but the majority fail to thrive shortly after birth. It is plausible that deficiencies resulting from low levels of Fxn^G127V^ protein hinder adequate metabolic adaptation at birth, when the neonate must make a transition from a continuous transplacental supply of glucose to a variable fat-based food supply via a series of coordinated metabolic and hormonal changes coupled with a dramatic increase in oxygen availability (Ward Platt and Deshpande, 2005). Extremely low levels of Fxn (WT or G127V) could potentially exaggerate iron sulfur cluster (ISC) deficiency, affect multiple ISC-dependent processes, including respiration (Brzóska et al., 2006), DNA replication and repair (Fuss et al., 2015) or ribosome maturation (Kispal et al., 2005), and curtail the survival of MUT mice in the crucial period after birth. Comprehensive studies will be necessary to assess developmental milestones and to determine the precise mechanism and timing of premature lethality in MUT mice. Owing to reduced representation of postnatal mutant animals, we focused our initial analyses on MEFs isolated from Fxn^G127V^ MUT mice, and full molecular and behavioral phenotyping of Fxn^G127V^ mice will be described elsewhere.

Molecular analysis of MUT MEFs revealed that the G127V mutation does not reduce *Fxn* mRNA levels but has a profound negative impact on immunodetectable Fxn protein levels. The G130V mutation was shown to reduce the solubility of purified recombinant human FXN proteins *in vitro* (Correia et al., 2008) or when exogenously expressed at high levels (Clark et al., 2017). However, our data indicate that the Fxn^G127V^ protein expressed from the endogenous locus is detectable exclusively in soluble fractions. Moreover, endogenous Fxn^G127V^ is localized to mitochondria, as is expected and observed for the Fxn^WT^ protein.

Depending on the FRDA model, different effects on the integrity (e.g. ultrastructure), number and function of mitochondria have been reported. For instance, reduced mitochondrial biogenesis and, subsequently, the number of mitochondria was reported in FRDA patient-derived cells, in FXN^WT^ KD control cells and in brain and skeletal muscle from the KIKO mouse model (Jasoliya et al., 2017; Lin et al., 2017a,b), whereas accumulation of damaged mitochondria was observed in FRDA induced pluripotent stem cell-differentiated cardiomyocytes and cardiac *Fxn* conditional knockout (cKO) mice (Hick et al., 2013; Perdomini et al., 2014; Vyas et al., 2012). Our results demonstrate a significantly increased frequency of mtDNA lesions in Fxn^G127V^ MUT MEFs, even at early passages, whereas an increased number of mitochondria is correlated with time in culture (i.e. with increasing passage). Elevated mitochondrial biogenesis could be a compensatory mechanism initiated by Fxn^G127V^ MUT cells to overcome the underlying load of damaged mitochondria. Indeed, this type of mechanism was previously proposed to explain similar results obtained using FRDA fibroblasts harboring homozygous repeat expansions (García-Giménez et al., 2011). In addition to total content, analyses of the dynamic state of mitochondria also provided insight into the networks formed over time in Fxn^G127V^ MUT MEFs. Although the mitochondrial networks formed in early passage MUT cells appear indistinguishable from those in WT cells, filament lengths in later passage MUT MEFs are significantly shorter than those of WT cells at matched passage. Increased fragmentation could serve as an independent indicator of excessive mitochondrial damage in late passage Fxn^G127V^ MUT MEFs.

Elevated mitochondrial content is one feature of senescent cells and is often accompanied by increased production of reactive oxygen species, reduced membrane potential and reduced respiratory coupling (Korolchuk et al., 2017), additional phenotypes that were revealed by our bioenergetic studies of early and late passage MUT MEFs. Extracellular flux analyses have so far been reported only recently in FRDA cellular models, namely in sensory neurons differentiated from induced pluripotent stem cells derived from patients harboring homozygous repeat expansions (i.e. chronically low FXN^WT^ expression) (Igoillo-Esteve et al., 2020) and an inducible FXN overexpression HEK293 cell line (i.e. short-term high FXN^WT^ expression) (Vannocci et al., 2018). Basal and maximal respiration and ATP production were significantly reduced in FXN^WT^-deficient sensory neurons (Igoillo-Esteve et al., 2020), and no beneficial effects were observed for these parameters upon FXN^WT^ overexpression (Vannocci et al., 2018). Expression of only Fxn^G127V^ protein was sufficient to maintain basal respiration at levels observed for WT MEFs at early and late passages.

Impaired glucose utilization and increased β-oxidation in FRDA patients was revealed by metabolic labeling of freshly isolated platelets (Worth et al., 2015), and lipid metabolism defects have been reported in various FRDA cellular and animal models (Tamarit et al., 2016). Our study on Fxn^G127V^ MUT MEFs also reveals altered endogenous fatty acid utilization, and our results could indicate that MUT MEFs depend on oxidation of endogenous FAs for energy production more than WT MEFs do, or that their available fatty acid pool is lower. The increased dependence of MUT mitochondria on fatty acid oxidation could also be a compensatory mechanism for impairments in glycolysis or other metabolic pathways and might be related to increased susceptibility to oxidative stress.

Some growth phenotypes typified by FXN^WT^-deficient cellular models, such as reduced proliferation and viability and increased sensitivity to oxidative stress (Carletti et al., 2014; Cotticelli et al., 2019; La Rosa et al., 2019; Loría and Diáz-Nido, 2015; Palomo et al., 2011), are observed in Fxn^G127V^ MUT MEFs. However, most of these studies report significantly increased apoptosis upon depletion of Fxn^WT^, whereas Fxn^G127V^ MUT MEFs instead undergo growth arrest and senescence, suggesting a residual function of the mutant protein that sustains cell viability. The ability of certain antioxidant agents to restore proliferation of MUT MEFs supports this hypothesis.

Activation of the NRF2 pathway as a therapeutic target in FRDA has gained attention because positive results of a phase 2 study for Omav treatment of FRDA patients were recently reported (NCT02255435) (La Rosa et al., 2020; Lynch et al., 2019). Increased NRF2 levels (mRNA and protein), in addition to the induction of NRF2 target gene expression, are observed after 24 h of treatment of FRDA fibroblasts with DMF, idebenone or Omav (Petrillo et al., 2019). Moreover, treatment of fibroblasts from FRDA patients with DMF was shown to increase *FXN* mRNA levels (Jasoliya et al., 2019), but treatment with Omav or idebenone had no effect on *FXN* gene expression (Petrillo et al., 2019). Finally, Omav treatment resolved several mitochondrial defects attributed to low FXN^WT^/Fxn^WT^ levels in FRDA fibroblasts and was protective against oxidative stress-induced cell death (Abeti et al., 2018). Likewise, our study indicates that activation of NRF2 signaling in cells expressing only the Fxn^G127V^ protein is protective against cell death, at least via Omav- and DMF-mediated actions. Omav and DMF both work to increase NRF2 levels and transcriptional activity by regulating its repressor, Kelch-like ECH-associated protein 1 (KEAP1), thereby preventing its degradation and augmenting its nuclear translocation (Probst et al., 2015; Takaya et al., 2012). We can speculate that the typical NRF2 antioxidant transcriptional response is hampered, but we cannot rule out the involvement and regulation of cell cycle protein expression, and therefore additional studies will be necessary to determine the mechanism(s) of action of DMF and Omav in enhancing the viability of Fxn^G127V^ MUT MEFs.

Our results demonstrate that cells expressing only the Fxn^G127V^ mutant protein exhibit many shared pathophysiological characteristics of cells expressing low levels of Fxn^WT^ protein, especially reduced growth potential and sensitivity to oxidative stress. However, some phenotypes, especially those pertaining to lipid handling, appear to be attributed uniquely to expression of the Fxn^G127V^ mutant protein. These results support a hypothesis of Fxn^G127V^/FXN^G130V^ proteins assuming alternative functions rather than loss of function. Changes in Fxn/FXN function induced by the G127V/G130V mutation, whether it be differential association with ISC assembly proteins or by eliciting new interactions, could help to shed light on unique molecular events underlying the atypical clinical presentation of FRDA G130V patients. It will be crucial to define functional relationships of the Fxn^G127V^ protein in neurons involved in prominent FRDA G130V symptoms, such as spastic paraparesis. Examining the effects of Fxn^G127V^ expression on disease-relevant tissues, such as those of the central nervous system and the heart, in the mouse model, and accounting for temporal aspects of development and aging processes are of immediate interest.

## MATERIALS AND METHODS

### Animals

Mice were housed in the animal facility at the University of Alabama at Birmingham under 12 h/12 h light/dark conditions and fed *ad libitum*. All experimental procedures were conducted in accordance with the Guide for the Care and Use of Laboratory Animals published by the US National Institutes of Health (NIH publication no. 85-23, revised 1996) and were approved by the Institutional Animal Care and Use Committee at the University of Alabama at Birmingham.

### Generation of Fxn G127V mice

The G127V knock-in mutation (GGC→GTC codon change) was introduced using CRISPR/Cas9 endonuclease-mediated genome editing via homologous-directed repair. Sequences for the guide RNA and oligonucleotide donor used are provided in Table S2. A silent D126D (GAT→GAC) was co-introduced with the G127V mutation as a PAM blocker. C57BL/6J zygotes with well-recognized pronuclei were microinjected with guide RNA, the mutation-bearing oligonucleotide donor and Cas9 nuclease. After injection, the embryos were transferred to pseudopregnant females. Two founders in 31 progeny were identified that contained the desired D126D, G127V mutations. Both were mated to C57BL/6J, and a single founder demonstrated transmission of the targeted D126D, G127V mutation through the germline. This mouse strain was designated JR 30822 (C57BL/6J-Fxn^em8Lutzy^/J). The population of JR 30822 mice was expanded with an additional backcross to C57BL/6J mice before intercrossing to score for the effect on homozygosity. The colony is maintained by backcrossing of heterozygous mice to wild type (C57BL/6J or wild-type littermates).

### Genotyping

Mouse genomic DNA was isolated from ∼3 mm tail biopsies with QuickExtract™ DNA Extraction Solution (Lucigen # QE09050) and used as a template for PCRs. A 300 bp fragment encompassing the *Fxn* point mutation was amplified with JumpStart™ Taq DNA Polymerase (Sigma-Aldrich, #D9307) using Fxn G127V restriction fragment length polymorphism primers found in Table S2. After amplification, the PCR product was digested overnight at 37°C with 0.5 μl of the restriction enzyme AatII (NEB, #R0117) added directly to the PCR tube. Subsequently, the reaction products were resolved by 1% agarose gel electrophoresis and visualized with ethidium bromide.

### Derivation and culture of MEFs

MEFs were derived according to (Jozefczuk et al., 2012). Briefly, pregnant female mice at 13 or 14 dpc were sacrificed and embryos extracted into Petri dishes. After removal of heads and red organs, the remaining tissues were minced, trypsinized with 0.05% trypsin/EDTA and incubated for 15 min at 37°C. Disrupted tissues were centrifuged at 300 ***g*** for 5 min, the supernatant was removed, and pelleted cells were suspended in Dulbecco's modified Eagle's medium (DMEM) high-glucose medium with pyruvate (Life Technologies, 11995) supplemented with 10% fetal bovine serum (HyClone, SH30396.03), 1% GlutaMAX (Life Technologies, 35051), and 1% penicillin-streptomycin (Life Technologies, 15140). Cells from each embryo were plated separately on gelatin-coated 100 mm dishes and propagated in DMEM (as above) supplemented with 10% fetal bovine serum and 1% GlutaMAX. Experiments denoted as ‘early passage’ were performed with cells between passage numbers 3 and 5, whereas ‘late passage’ denotes cells of passages 6-9. MEF cell lines derived from at least two different embryos per genotype were used for each experiment, and considered as independent biological replicates.

### Treatments and XTT assays

WT and MUT MEFs were seeded at 20,000 cells per well in 96-well plates 24 h before treatments. Compounds were added and cells incubated for an additional 24 h, followed by incubation with XTT [(sodium 2,3,-bis(2-methoxy-4-nitro-5-sulfophenyl)-5-[(phenylamino)-carbonyl]-2H-tetrazolium)] reagent for 3 h. The XTT assays were performed as recommended by the manufacturer (ATCC). Commercially available compounds were obtained as follows: Omav (RTA 408; Cayman Chemical), DMF (Tocris) and idebenone (Sigma-Aldrich).

### Quantitation of mRNA

RNA was isolated from MEFs using the RNeasy Mini Kit (Qiagen, #74104). Genomic DNA contamination was removed with the TURBO DNA-free™ Kit (Invitrogen, #AM1907). The level of specific mRNA was quantitated by Power SYBR™ Green RNA-to-CT™ 1-Step Kit (Applied Biosystems, #4389986). Transcript (mRNA) levels relative to that of the housekeeping gene glyceraldehyde phosphate dehydrogenase (*Gapdh*) were calculated using the ΔΔ*Ct* method. Primer sequences can be found in Table S2.

### Western blot

For preparation of total protein lysates, MEFs were homogenized in NP-40 lysis buffer containing 1% NP-40, 20 mM Tris-HCl pH 7.4, 150 mM NaCl, 5 mM EDTA, 10% glycerol, 1 mM dithiothreitol, 10 mM sodium fluoride, 1 mM sodium orthovanadate and 5 mM sodium pyrophosphate, with protease inhibitor cocktail (Millipore, #539134). The lysates were rotated for 30 min at 4°C, followed by centrifugation at 15,800 ***g*** for 20 min. The supernatant was removed as the soluble fraction. To reduce carryover, the pellets were washed with lysis buffer and resuspended in urea-sodium dodecyl sulfate buffer (NP-40 lysis buffer with 8 M urea/3% sodium dodecyl sulfate) followed by sonication, with three 20 s pulses at 20% amplitude on a Fisher Scientific™ Model 120 Sonic Dismembrator. The lysates were then centrifuged again at 15,800 ***g*** for 20 min at 4°C and the supernatant was collected as the insoluble fraction. Protein concentration of soluble fractions were estimated by Bradford assay using Protein Assay Dye Reagent (Bio-Rad, #5000006). Samples were heated with NuPAGE™ LDS Sample Buffer (4×) (Invitrogen, #NP0008) and NuPAGE^®^ Reducing Agent (10×) (Invitrogen, #NP0004) for 10 min at 70°C. Protein samples were resolved on a 4-12% NuPAGE gel and transferred to nitrocellulose membranes with an iBlot™ 2 Gel Transfer Device (Invitrogen, #IB21001). Membranes were blocked with 5% ECL™ Advance Blocking Reagent (GE Healthcare, #RPN418) followed by incubation with primary and secondary (anti-rabbit horseradish peroxidase linked, GE Healthcare, #LNA934V) antibodies. Antibody information is provided in Table S3. For enhanced sensitivity, membranes were incubated with SuperSignal™ Western Blot Enhancer (Thermo Fisher Scientific, #46640) for 10 min at room temperature (RT) before blocking, and high-intensity horseradish peroxidase substrate SuperSignal™ West Femto (Thermo Fisher Scientific, #34094) was used to produce a signal. All images were captured on a ChemiDoc MP Imaging System and analyzed using ImageLab v.6.0.1 software (Bio-Rad).

Mitochondrially enriched protein lysates were prepared as described (Lin et al., 2017b), with modifications. Cells were resuspended in hypotonic buffer [225 mM mannitol, 75 mM sucrose, 5 mM Hepes, 1 mM EGTA, 0.1 mM EDTA (pH 8), 0.1% bovine serum albumin (BSA), 1% protease inhibitor cocktail (PIC; Sigma-Aldrich, P8340)], kept on ice for 10 min, then homogenized. The suspensions were centrifuged at 1500 ***g*** for 4 min at 4°C, after which supernatants were transferred to fresh tubes, and the suspensions were centrifuged again at 20,000 ***g*** for 15 min at 4°C. Supernatants were collected as ‘cytoplasmic’ fractions, and pellets were washed with buffer [50 mM Tris (pH 7.5), 0.25 M sucrose, 0.2 mM EDTA (pH 8), 0.1% BSA and 1% PIC], then resuspended in lysis buffer [0.1% NP-40, 0.25 M NaCl, 5 mM EDTA, 50 mM Hepes (pH 7.5) and 1% PIC] and kept on ice for 10 min for protein extraction. Lysates were centrifuged at 20,000 ***g*** for 10 min at 4°C, and clarified supernatants were transferred to fresh tubes as ‘mitochondrially enriched’ fractions.

### Growth curves and population doubling time of MEFs

Early passage WT, HET and MUT MEFs were seeded in duplicate in six-well plates at 1×10^5^ cells per well and cultured in either a cell culture incubator with ‘normoxic’ gas composition (air supplemented with 5% CO_2_) or ‘hypoxic’ gas composition (90% N_2_, 5% CO_2_ and 5% O_2_). Cells were detached with trypsin and counted daily after seeding (days 1-6). The PDT was calculated during the logarithmic phase of the growth using the formula: PDT=*T*×ln2/ln(*A*/*A*_0_), where *T* corresponds to the time duration of culture (in hours), *A* corresponds to the number of cells in the well at the time of measurement and *A*_0_ is the initial number of cells.

### Measurement of cell death and cell cycle

MEFs were plated at 1×10^5^ WT or 2×10^5^ MUT cells per 100 mm dish and cultured until ∼80% confluent. Cells were detached with accutase and washed with PBS. For cell death analysis, MEFs were processed with the FITC Annexin V Apoptosis Detection Kit I (BD BioSciences, #556547) according to the manufacturer's instructions. For cell cycle analysis, MEFs were fixed with cold 70% ethanol overnight, then stained with PBS supplemented with Triton X-100 (final concentration 0.1%), RNase A (final concentration 200 µg/ml) and PI (final concentration 20 µg/ml) for 30 min at RT. Stained MEFs were counted with an LSRII Analyzer, BD Diva v.8.0.1 (BD BioSciences) and data analyzed with the FlowJo software package using a Watson pragmatic model.

### Senescence-associated β-galactosidase staining

Cell staining was performed with a Senescence β-galactosidase Staining Kit (Cell Signaling Technology, #9860), following the manufacturer's instructions. At least ten images were taken per group at ×200 total magnification, and the fraction of blue-stained cells counted by a blinded investigator. To avoid staining attributable to cell confluence rather than to proliferative senescence (Severino et al., 2000), the assay was performed in subconfluent cultures displaying comparable cell densities.

### Staining of mitochondria

MEFs were plated on glass coverslips, cultured until ∼60-80% confluent and stained with OptiMEM supplemented with 100 nM (final concentration) MitoTracker™ Deep Red FM (Thermo Fisher Scientific, #M22426) for 30 min at 37°C followed by 30 min incubation in OptiMEM without the dye. Stained MEFs were fixed with 3.7% formaldehyde for 15 min at 37°C, mounted on microscope slides with ProLong™ Gold Antifade Mountant with 4′,6-diamidino-2-phenylindole (Invitrogen, #P36935) and imaged with a Nikon A1R confocal microscope using ×63 objective magnification (University of Alabama at Birmingham High Resolution Imaging Facility). Maximum-intensity projections of confocal image *z*-stacks were rendered as three-dimensional reconstructions using Imaris analytical software (Bitplane AG). Imaris Filament Tracer tool with subjective thresholding was used to detect mitochondrial filament lengths automatically.

### Mitochodrial DNA damage and copy number in MEFs

Mitochondrial DNA damage was determined by quantitative PCR (qPCR) as described by Furda et al. (2014), with some modifications. Briefly, total DNA was isolated with the QIAamp DNA Mini Kit (Qiagen, #51304) according to the manufacturer's instructions. Long mtDNA fragments were amplified with high-fidelity AccuPrime™ Taq DNA polymerase (Thermo Fisher Scientific, #12346086) and quantified with the Quant-iT™ PicoGreen™ dsDNA Assay Kit (Thermo Fisher Scientific, #P7589). A short mtDNA fragment was quantified with Power SYBR™ Green PCR (Thermo Fisher Scientific, #4367659) relative to genomic DNA. Quantitation of the average lesion frequency in mtDNA was performed assuming a random distribution of lesions in accordance with the Poisson equation (Westbrook et al., 2010). The mtDNA copy number was estimated by determination of the mtDNA to nuclear DNA ratio with a qPCR assay as described by Quiros et al. (2017). Primer sequences can be found in Table S2.

### Measurement of mitochondrial respiration

The mitochondrial function of MEFs was determined using a Seahorse Extracellular Flux Analyzer (XF96 Analyzer; Agilent Technologies, Santa Clara, CA, USA), which measures the OCR in live cells. Cells were plated at 40,000 cells per well in a XF96 assay plate and allowed to attach for 4 h in culture medium before the assay, washed using XF-DMEM assay medium and allowed to equilibrate in a non-CO_2_ incubator for 1 h at 37°C. Mitochondrial stress tests were performed by sequentially injecting oligomycin (Oligo, 1 µg/ml), carbonyl cyanide 4-(trifluoromethoxy)phenylhydrazone (FCCP, 1.5 µM) and antimycin A (Anti A, 10 µM) (Chacko et al., 2013). To record fatty acid-mediated OCR measurements, BSA-palmitate substrate (25 µg/ml) was incubated with MEFs for 30 min before the assay in XF-DMEM medium (Ravi et al., 2015). Etomoxir (10 µM) was added to determine fatty acid-sensitive OCR (Ravi et al., 2015). The data were normalized to the total protein content in each well, measured using the Lowry HS protein assay (Bio-Rad) and expressed as the mean.

### Statistical analyses

Statistical analyses were conducted using GraphPad Prism v.6. Statistical significance was determined by performing Student's unpaired two-tailed *t*-tests, multiple *t*-tests (with Holm–Sidak correction for multiple comparisons), ordinary one-way ANOVA or two-way ANOVA (with Tukey correction for multiple comparisons). Significant differences were considered as *P*<0.05.

## Supplementary Material

Supplementary information
