# Supplementary material for: Mitochondrial damage and senescence phenotype of cells derived from a novel frataxin G127V point mutation mouse model of Friedreich's ataxia
Source: Dis Model Mech. 2020 Jul 27;13(7):dmm045229. doi: 10.1242/dmm.045229 (PMC7406325; doi:10.1242/dmm.045229)
Supplement: Supplementary information [file dmm-13-045229-s1.pdf]

SUPPLEMENTARY INFORMATION

Mitochondrial damage and senescence phenotype of cells derived from a novel frataxin G127V point mutation mouse model of Friedreich's ataxia

Daniel Fil[1], Balu K. Chacko[2,3,4], Robbie Conley[1], Xiaosen Ouyang[2,3,4,5], Jianhua Zhang[2,3,4,5], Victor M. Darley-Usmar[2,3,4], Aamir R. Zuberi[6], Cathleen M. Lutz[6], Marek Napierala[1], Jill S. Napierala[1]*

[1]Department of Biochemistry and Molecular Genetics, University of Alabama at Birmingham, 1825 University Blvd, Birmingham, AL 35294, USA
[2]Department of Pathology, University of Alabama at Birmingham, 901 19th Street South, Birmingham, AL 35294, USA
[3]Center for Free Radical Biology, University of Alabama at Birmingham, Birmingham, AL 35294, USA
[4]Mitochondrial Medicine Laboratory, University of Alabama at Birmingham, Birmingham, AL 35294, USA
[5]Department of Veteran Affairs Medical Center, Birmingham, AL 35294, USA
[6]The Rare and Orphan Disease Center, JAX Center for Precision Genetics, 600 Main Street, Bar Harbor, ME 04609, USA

*To whom correspondence should be addressed
Correspondence should be addressed to: Jill S. Napierala, University of Alabama at Birmingham, Department of Biochemistry and Molecular Genetics, 1825 University Blvd., Birmingham, Alabama 35294, USA. (205) 975 5320 phone, (205) 975 3335 fax, jsbutler@uab.edu

Disease Models & Mechanisms • Supplementary information

Fig. S1. Gross phenotypic analysis of WT, HET, and Fxn^{G127V} MUT late stage embryos.
Three litters of late-stage embryos at E20 and E19 (*n*=24 embryos) were isolated, weighed, visually assessed, and genotyped. Representative images are shown for WT, HET, MUT; Scale bar, 1 cm. Weights relative to the average weight of WT embryos per each litter are presented as box and whisker plots with medians shown as horizontal lines and boxes extending from the 25th to 75th percentiles. Whiskers extend to minimum and maximum values and each embryo is represented by an individual dot. Significant differences are indicated by asterisks as determined by one-way ANOVA analysis using Tukey's method for multiple comparisons (*** *P*<0.001, **** *P*<0.0001).

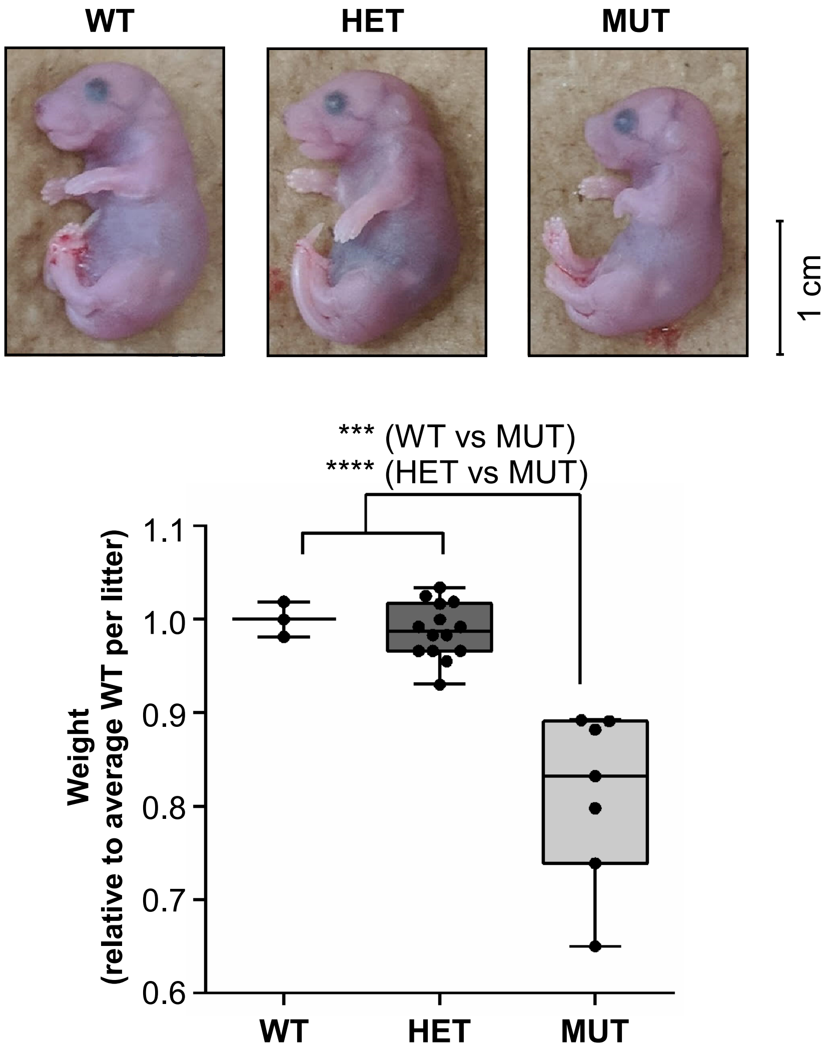

Disease Models & Mechanisms • Supplementary information

Fig. S2. Validation of anti-frataxin antibodies for detection of Fxn^{G127V} protein.

A) Fxn-WT-FLAG and Fxn-G127V-FLAG proteins transiently overexpressed in HEK-293T cells were detected by western blot using three different commercially available anti-Frataxin antibodies. B) The Genetex antibody (GTX54036; left panel A) showed reduced sensitivity toward endogenous Fxn^{G127V} protein when used for western blot analysis of MEF lysates, even when enhanced sensitivity western blotting techniques were used. Therefore, the Proteintech antibody (PTG14147; middle panel) was selected for use to probe MEF lysate western blots (Fig. 2).

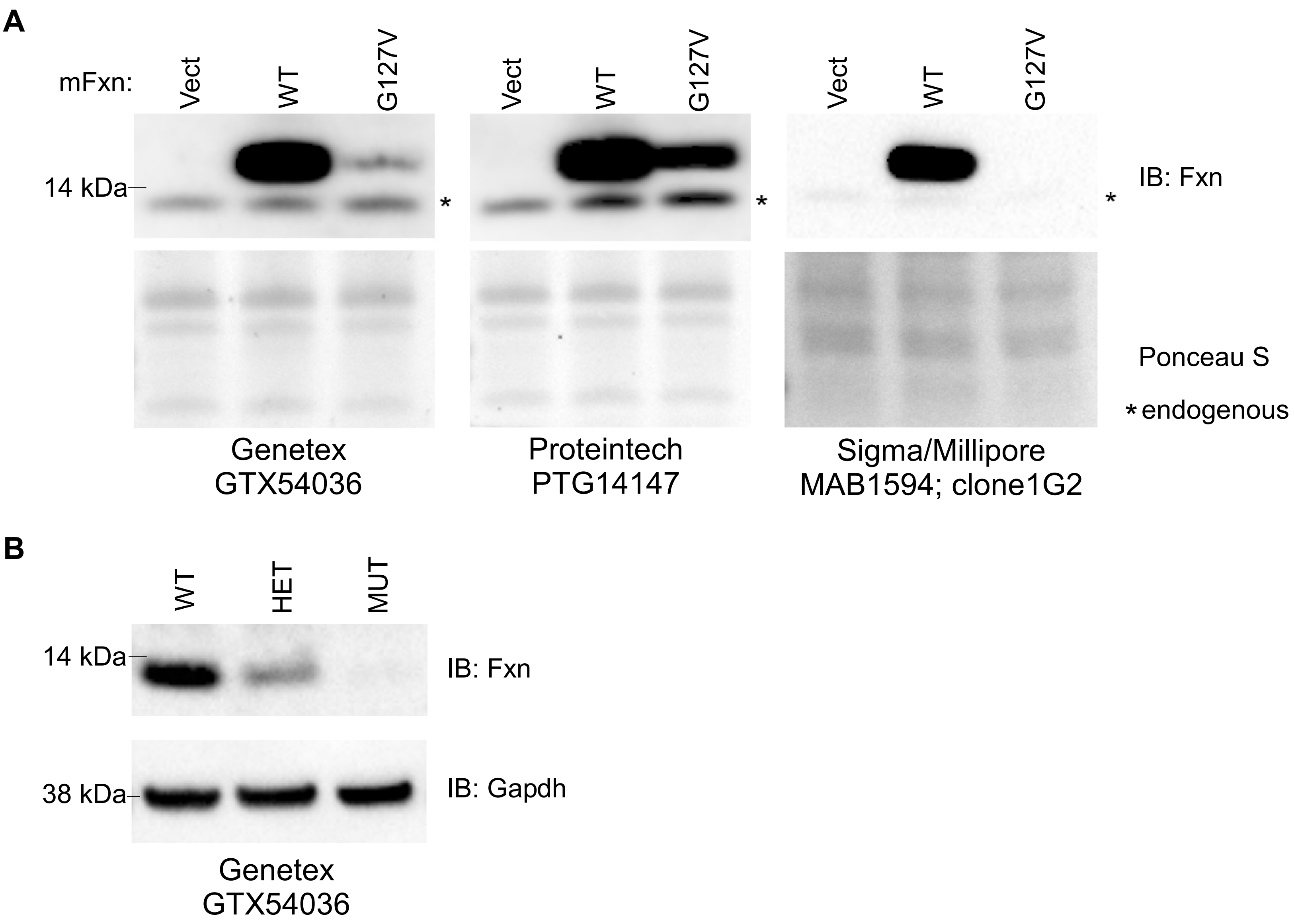

Disease Models & Mechanisms • Supplementary information

Fig. S3. The propensity of Fxn^{G127V} MUT MEFs to senesce increases with passage number.
Quantification of cells staining positive for SA-β-galactosidase (expressed as a percentage of total cells counted) in WT and MUT MEF cultures (*n*=2 biological replicates/genotype) at sequential passages. Bars are mean±s.d. of at least two independent experiments; total measurements/bar=4-19. Asterisks denote significant differences as determined by unpaired Student's *t*-tests at each passage (**** *P*<0.0001).

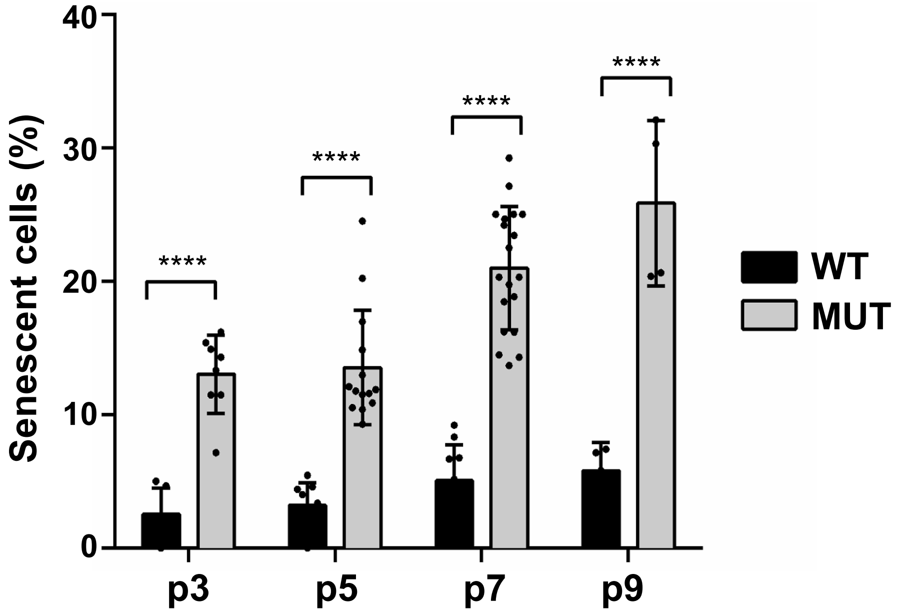

Disease Models & Mechanisms • Supplementary information

Table S1. Analysis of Fxn G127V mutant allele transmission.

**N2**	**WT**	**HET**	***total***
Female	21	18	*39*
Male	27	14	*41*
total	*48*	*32*	*80*
%	60	40	

Table S2. Sequences of primers and other oligonucleotides used in these studies.

**Primer**	**Forward 5 → 3**	**Reverse 5 → 3**
	Genotyping	
Fxn G127V RFLP	CATTACTCTCAGGGTCAGAATGGGTCAG	CCCAACACCGCATTCCAGATGAGTT
	Quantitative RT-PCR for Fxn mRNA	
Fxn RT1	GTCTGCGTGGTGCATTTGAG	AGGGAGTCCAGGGTCTCTTC
Fxn RT2	ACAAGCAGACCCCAAACAAG	GACACGCCGTCATGAGAGTA
Fxn RT3	ACCTCGCAGACAAGCCCTAT	GATCCCCGCCCAGCTTAATG
Gapdh	AGGTCGGTGTGAACGGATTTG	TGTAGACCATGTAGTTGAGGTCA
	mtDNA damage	
Long mtDNA (10085 bp)	GCCAGCCTGACCCATAGCCATAATAT	GAGAGATTTTATGGGTGTAATGCGG
Short mtDNA (117 bp)	CCCAGCTACTACCATCATTCAAGT	GATGGTTTGGGAGATTGGTTGATGT
Genomic DNA (*Hk2* gene; hexokinase 2)	GCCAGCCTCTCCTGATTTTAGTGT	GGGAACACAAAAGACCTCTTCTGG
	mtDNA copy number	
Genomic DNA	GCCAGCCTCTCCTGATTTTAGTGT	GGGAACACAAAAGACCTCTTCTGG

Disease Models & Mechanisms • Supplementary information

(*Hk2* gene; hexokinase 2)		
mtDNA (*mt-Rnr2* gene; 16S rRNA)	CCGCAAGGGAAAGATGAAAGAC	TCGTTTGGTTTCGGGGTTTC
CRISPR guide and donor sequence		
sgRNA	TGCCACCTGACCCCCTAGGA	
ssODN	AGAAGACAGCCAGATTTGCTTGTTTGGGGTCTGCTTGTTGATCACGTAGGTCCCT AGATCCCCGCCCAGCTTAATGGTGAGCACGACGTCCTAGGGGGTCAGGTGGCAG GAAGTGTGAAAAAATAGATTAGTTAG	
Fxn cDNA cloning and sequencing		
mFxn ex1 F	GGAGTAGCATGTGGGCGTT	
mFxn ex5 R		ACACGGTGTGACTATCTTCAGG

Disease Models & Mechanisms • Supplementary information

Table S3. Information for antibodies used in these studies.

Antibody	Vendor	Catalog number
Frataxin	Proteintech	14147-1-AP
Frataxin	Genetex	GTX54036
Frataxin	Sigma/Millipore	MAB1594; clone 1G2
Hprt	Proteintech	15059-1-AP
Gapdh	Millipore	MAB374
Nfs1	Santa Cruz Biotechnology	sc365308
Iscu	Santa Cruz Biotechnology	sc373694

Disease Models & Mechanisms • Supplementary information
